# Nanomaterial Technology and Soft Tissue Sarcomas

**DOI:** 10.3389/fonc.2022.921983

**Published:** 2022-06-22

**Authors:** Changkai Zhou, Xue Chen, Ying Huang, Qi Zhang, Shu Zhu, Wei Fu

**Affiliations:** ^1^ Department of Burn and Plastic Surgery, Affiliated Hospital 2 of Nantong University, Nantong First People’s Hospital, Nantong, China; ^2^ Department of Plastic and Cosmetic Surgery, Tongji Hospital, Tongji Medical College, Huazhong University of Science and Technology, Wuhan, China; ^3^ Department of Operation Room, Affiliated Hospital 2 of Nantong University, Nantong First People’s Hospital, Nantong, China; ^4^ Department of Medical Ultrasound, Tongji Hospital, Tongji Medical College, Huazhong University of Science and Technology, Wuhan, China; ^5^ Department of Pharmacy, Tongji Hospital, Tongji Medical College, Huazhong University of Science and Technology, Wuhan, China

**Keywords:** soft tissue sarcoma, nanotechnology, cancer diagnosis, drug delivery, photodynamic therapy, radiotherapy

## Abstract

Soft tissue sarcomas (STSs) are relatively rare heterogeneous solid tumors of the mesenchymal origin. They account for approximately 1% of all malignant tumors in adults and have more than 70 histological subtypes. Consequently, the rarity and heterogeneity of STSs make their diagnosis and treatment very challenging. Nanotechnology has attracted increasing attention from researchers due to the unique physicochemical and biological properties of nanomaterials with potential medical applications as nanoprobes, drug delivery systems, photosensitizers, radioenhancers, antitumor agents, and their combinations for cancer diagnosis and treatment. This review discusses the progress made in the use of nanotechnology for the diagnosis and treatment of STSs and highlights future prospects of the STS multimodality therapy.

## Introduction

Soft tissue sarcomas (STSs) are rare and heterogeneous tumors representing only 1% of all adult malignancies and encompassing more than 70 histological subtypes with variable clinical behavior (1). It is estimated that in 2022, 13,190 new STS cases will be diagnosed in the USA, causing approximately 5,130 deaths (2). These malignant mesenchymal tumors are important and often overlooked causes of death in young patients. According to the American Cancer Society, the average overall five-year STS survival rate is approximately 65% (2). Prognostic prediction accuracy strongly depends on the tumor subtype, size, grade, stage, and response to treatment as well as demographic factors. However, the lack of known risk factors and appropriate preoperative preparation may delay STS diagnosis (3). Thus, referrals often originate from the results of the so-called “whoops” procedures (4). Currently, histologic grade is recognized to be the most important prognostic factor predictive of distant metastasis and disease-specific survival rate (5). The traditional treatment of patients diagnosed with STS includes surgical resection, radiotherapy, chemotherapy, photodynamic therapy, and their combinations, which are slowly evolving (6). Therefore, it is necessary to investigate effective methods of improving the efficiency of STS diagnosis and treatment.

Nanomaterial technology has attracted significant attention from researchers over the years and tremendous progress has been made in this field. The unique physical and functional properties of nanomaterials make them potentially suitable for the management of many diseases, including cancer (7). Therefore, nanomaterials are widely used for cancer diagnosis, monitoring, imaging, and treatment. For example, various nanovehicles for the efficient delivery of drugs, such as liposomes, micelles, dendrimers, quantum dots, and carbon nanotubes, have been developed (8, 9). Additionally, nanoparticles with certain sizes can convert light into heat, which causes cellular damage during photothermal therapy. Nanoparticles with high electron density and well-defined size and shape are highly efficient at absorbing radiation and, therefore, can be used as radioenhancers in radiotherapy (10). Moreover, a direct antitumor effect was observed for certain nanoparticles (11). Considerable progress has been made in the application of nanotechnology for STS diagnosis and treatment. This review describes the latest developments in the use of nanomaterials for STS diagnosis and treatment, highlighting the future prospects of STS multimodality therapy.

## Diagnosis and Subtypes of STSs

STSs tends to form as a large, painless, and unexplained mass with a consistently increasing volume, which is the best individual indicator of a high malignancy risk (12). Therefore, patients suspected of having STS (especially those having superficial lesions with sizes greater than 5 cm) should initially undergo an ultrasound examination, which is cost-effective and has a high negative predictive value for soft tissue lumps (13). Magnetic resonance imaging (MRI) has a very high negative predictive value in distinguishing lipomatous tumors (14) and is helpful during surgical and radiotherapy planning. Contrast-enhanced computed tomography is an alternative imaging modality for patients with contraindications to MRI, which plays an important role in the evaluation of lungs for metastatic sarcomas. Moreover, core needle biopsy provides a basis for definitive tissue diagnosis, which is critical for cases, in which neoadjuvant therapy is administered (15). Molecular genetic testing plays an increasingly important role in the classification of STSs (16, 17). Therefore, proper sarcoma diagnostics requires a multidisciplinary approach with the participation of an expert team of pathologists, radiologists, medical oncologists, molecular biologists, and surgical oncologists (18).

Most STSs are classified according to their tissue origin and differentiation characteristics. Some of them are named based on their histological patterns (e.g., soft pulmonary alveolar sarcoma and epithelioid sarcoma). Unfortunately, STSs have over 70 histological subtypes (1, 19). Among those, the three most common histologic subtypes of STSs in adults include undifferentiated pleomorphic sarcoma (UPS), liposarcoma, and leiomyosarcoma. Histological diagnosis should be based on the 2020 World Health Organization classification of soft tissues and bone tumors (1). The grading system proposed by the French Federation of Cancer Centers Sarcoma Group is most widely used due to its high reproducibility and precise definitions (20, 21). Unlike other cancers, STSs have a high prognostic value according to the American Joint Committee on Cancer TNM staging system owing to the inclusion of histological grading. In summary, the rarity and heterogeneity of STSs make its diagnostic a very complex and difficult process.

## Management of STSs

Owing to the rarity and heterogeneity of STSs, patients with STSs should be referred to specialized sarcoma centers for curing or palliative care depending on the STS grade (18, 22). First, radical surgical resection is the cornerstone of the treatment of primary STSs. Notably, an adequate preoperative workup, including advanced imaging studies and diagnostic biopsy, should be performed to eliminate the risk of incomplete STS excision (3). In other words, the key principles of surgery include oncological radicality and function sparing. For extremity and truncal STSs, at least 1–2 cm of the healthy tissue around the tumor must be removed. Moreover, for the highly invasive histological subtypes such as locally invasive myxofibrosarcoma, the resection margin should be at least 3 cm (23). However, amputation is a better choice when the tumor involves the major vessels and nerves in the limbs. Fortunately, due to the progress in neoadjuvant treatment, the rate of limb salvage is constantly increasing (24). Resections were classified into R0 (with microscopically negative margins), R1 (with microscopically positive margins), and R2 (with macroscopically positive margins) according to the results of postoperative pathological examination (25). However, R0 resections are sometimes difficult to perform due to anatomic constraints (i.e., retroperitoneal sarcomas, which represent approximately 15% of all STSs). Thus, (neo)adjuvant therapies should be considered to improve prognosis.

Most low-grade and well-edged STSs do not require adjuvant radiotherapy, whereas for the moderate-to-high grade STSs, adjuvant radiotherapy may improve the local control and prognosis (26). Additionally, radiotherapy plays a significant role in the palliative or definitive treatment of patients who cannot undergo R0 resection. Although the effect of radiotherapy on limb STSs is clear, the optimal radio-surgical strategy remains controversial (27–30). However, with the recent advances in radiation techniques, including the use of nanosized radioenhancers (such as hafnium oxide nanoparticles, NBTXR3), the cost/benefit ratio of radiotherapy has considerably decreased (31, 32). Chemotherapy is the first option for metastatic and locally advanced STSs, and the most commonly used drugs include anthracyclines, gemcitabine, and taxanes (33). Nevertheless, the heterogeneity of various STS subtypes often results in different responses to chemotherapeutic agents. Many studies were performed to explore the role of adjuvant chemotherapy in STS treatment, but their results were contradictory, and the benefits of chemotherapy remained uncertain (34–37). Fortunately, advances in nanosized drug delivery systems may overcome the main drawbacks of traditional chemotherapy, including low efficacy and toxicity. Over the years, significant progress has been made in the field of targeted therapy for STSs (38, 39). Tyrosine kinase inhibitors (TKIs), such as imatinib, sunitinib, and regorafenib, were approved for the treatment of gastrointestinal stromal tumors (GISTs) (40–43). Pazopanib, another TKI, was approved for the treatment for non-GIST STSs (44). Olaratumab, a monoclonal antibody, was approved for the treatment of STSs (45). However, even the best formulation of neo-adjuvant therapies cannot replace R0 resection (46).

## Nanotechnology and STSs

Owing to the rarity and multiplicity of clinical behavior, STS diagnosis is sometimes delayed, and the treatment of STSs becomes a complex process; as a result, the prognosis is usually dismal. Therefore, more efforts should be spent on exploring new options for the diagnosis and treatment of STSs. Nanotechnology has attracted scientific interest owing to its various advantages, including desirable bioavailability, specialized structures, and promising drug encapsulation efficiency. Moreover, significant progress has been made in the field of nanomaterials, which exhibit unique physical and chemical properties and are widely used in the health and medical fields (47). The application of nanomaterials offers potential advantages in the diagnosis and treatment of many diseases, including cancer (7, 48, 49). For example, nanomaterials can be used to detect tumor markers for the purpose of diagnosis and evaluating prognosis (50). Drug-loaded nanomaterials may cross many biological barriers and be transported to a target region (51). Additionally, nanomaterials play an important role in photodynamic therapy and radiotherapy.

### Nanoprobe and STSs

Early detection is associated with timely treatment and, hence, better prognosis. This is particularly true for STSs, which are often overlooked by the general public and healthcare providers. Traditional diagnostic strategies have various limitations, such as the low sensitivity of imaging techniques and unnecessary contamination of healthy tissues due to a poorly performed biopsy. Researchers are currently exploring potential breakthrough points for tumor diagnostic technologies. Thus, the role of nanoparticles in cancer diagnosis and surveillance has attracted increasing attention over the past few decades due to their intrinsic magnetic, optical, and electrical properties (48). Additionally, nanoprobes coupled with specific ligands can accumulate at the tumor site or generate high responses to very small targets *via* ligand-receptor interactions to produce signals for ultrasensitive detection (50).

Tumor formation is caused by the uncontrolled cell division due to mutations in specific genes that alter the synthesis of specific biomolecules. The overexpression of tumor-associated proteins (TAPs) occurs during tumor formation, and TAPs can regulate proteolysis, making them an important factor in cancer progression. For example, MMP-2 is a TAP overexpressed in most solid tumors including STSs (52), which can be used for the detection of such tumors. Wang et al. (53) developed a nanoprobe containing an MMP-2 substrate sequence for detecting MMP-2-overexpressed tumors, which is highly activated in human fibrosarcoma HT1080 cells *in vitro* and highly expressed in human fibrosarcoma HT1080 cell xenografts compared with MCF-7 cells. Their study demonstrates the sensitivity and specificity of this prodrug-type nanoprobe for tumor detection and imaging. Nevertheless, the heterogeneity of specific biomarkers in each STS subtype makes the development of a foolproof strategy for STS detection a challenging task.

### Drug Delivery Nanosystems and STSs

Despite the large number of drugs available for cancer treatment, conventional chemotherapeutic agents have various side effects, such as non-specific distribution, low bioavailability, toxic effects on healthy cells, and resistance development. Therefore, advanced drug delivery systems should be created overcome these drawbacks. Recent developments in nanotechnology-based drug delivery systems (DDSs) are expected to improve the drug delivery process and control drug release using passive or active targeting strategies, thereby reducing the side effects of chemotherapy during tumor treatment. In addition, they can deliver multiple drugs simultaneously to perform a combination therapy (54). Over the years, a large number of nanomedicines have been used to assess their potential for the treatment of STSs, including the balance between efficacy and toxicity (**Table 1**).

**Table 1:** Nanomaterials used in the preclinical research of STS treatment.

Nanomaterial	Effect	Target	Cargo	Tumor Cell line/ animal model	Observation	Reference
Glucosylated polymeric nanomicelles	Active targeting	GLUT-1	dasatinib	RMS cell line Rh30. Patient-derivedglucose-avid Rh30 xenograft	In vitro: a 9-fold decrease of the half maximal inhibitory concentration of dasatinib in a RMS cell line, Rh30. In vivo: selective accumulation of dasatnib in a patient-derived RMS model	(55)
LPR nanoparticles	Active targeting	PAX3-FOXO1 (P3F)	siRNA	Human RMS cell lines. Rh30 cells xenograft	Significant tumor growth delay and inhibition of tumor initiation	(56)
4-arm-PEG_5K_-TPGS nanoparticles	Passive targeting	/	Paclitaxel	S180 sarcoma-bearing mice	Significantly improved tumor growth inhibitory effect	(57)
CP nanoparticles	Passive targeting	/	Doxorubicin	MPNST and UPS sarcoma mouse models	CP-Dox formulation was superior to free doxorubicin in MPNST models, but not in UPS models.	(58)
Cerium oxide nanoparticles	Anticancer effect	/	/	Murine fibrosarcoma cells (WEHI164)	Significant toxicity on WEHI164 cells comparing with L929 cells. ROS generation in the cancer cells but scavenges it in the normal L929 cells.	(59)
	Anticancer effect	/	/	Mice bearing WEHI164 cells (mouse fibrosarcoma cells)	Dominantly accumulation in tumor cells. Significantly decrease tumor growth and volume.	(11)
Gold nanoparticles	Antimigration effect	/	/	Human fibrosarcoma cancer cell line HT-1080	No toxic effects on HT-1080 cells proliferation; Inhibition of cell migration	(60)
	Passive targeting	/	N-aminobacteriopurpurinimide (photoenhancer)	Rats bearing sarcoma M 1	Extended circulation time in the blood and enhanced tumor uptake	(61)
AuNRs/mSiO_2_	Multy modal phototherapy	/	ICG-Der-02	HT-1080 human fibrosarcoma cells	More damaging to HT-1080 cells and enhanced the effectiveness of photodestruction	(62)

**Figure 1:**
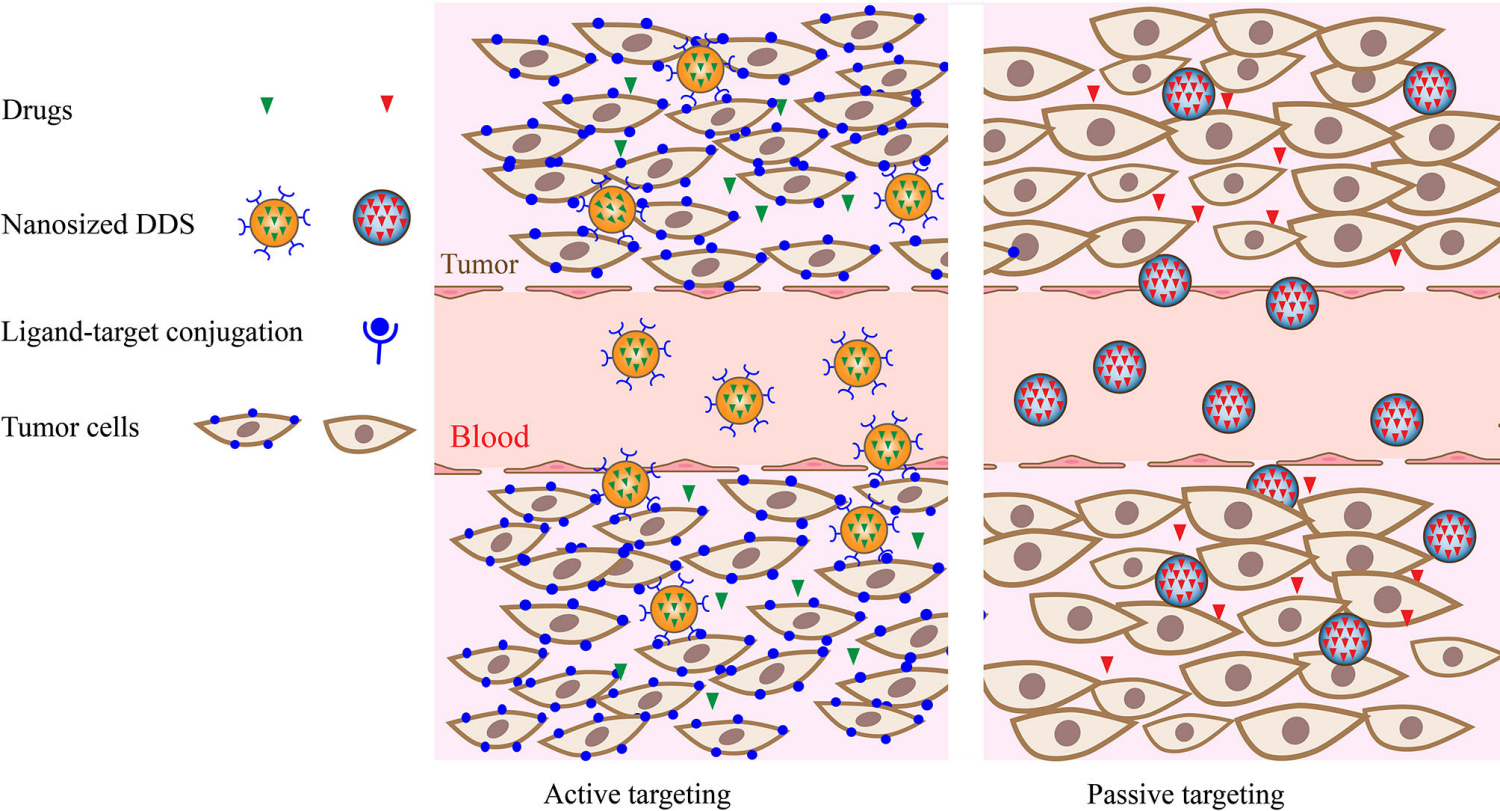
Active and passive targeting strategy of drug delivery nanosystems.

Active targeting relies on ligand–receptor binding to deliver drugs to cells and decrease nonspecific interactions in peripheral tissues (**Figure 1**). These high-affinity ligands may include aptamers, proteins, transferrin, antibodies, and other macromolecules that are specifically overexpressed on the surface of tumor cells. For example, the overexpression of glucose transporters (GLUTs) in pediatric sarcomas was used for active targeting. A nano-drug delivery system based on glycosylated polymer nanocapsules targeting dasatinib was reported for the first time by Bukchin et al. (55) In their study, the use of a glycosylated amphiphilic nanocarrier promoted the delivery of dasatinib in tumor parenchyma and reduced its accumulation in the off-target tissues and organs of immunodeficient mice bearing glucose-avid Rh30 xenograft. Additionally, small molecules, such as folic acid, carbohydrates, and nucleic acids, were identified. In another approach utilizing the active targeted delivery of nano-drugs, integrin receptor-targeted lipid protamine-siRNA (LPR) nanoparticles were developed and used to load siRNA targeting the PAX3-FOXO1 (P3F) breakpoint, which was a specific fusion transcript of alveolar rhabdomyosarcoma (ARMS) (56). The results of both *in vitro* and *in vivo* studies verified the specificity and efficacy of this novel therapeutic strategy. However, the lack of universal STS-specific targets may produce barriers for nanomedicine development due to the heterogeneity of STSs.

Passive targeting involves the diffusion-mediated transport of drugs(**Figure 1**). The enhanced permeability and retention (EPR) effect promotes the passive accumulation of nanodrugs in tumor cells. Thus, the efficiency of the passive targeting strategy is dramatically affected by the inherent properties of drug–carrier complexes, such as molecular size, weight, and surface hydrophilicity. For instance, paclitaxel (PTX) exhibits low aqueous solubility, which limits its delivery to tumors. To overcome this problem, Wu’s research group synthesized a novel nanocarrier, 4-arm-PEG_5K_-TPGS NP, to load PTX for further studies. The S180 sarcoma-bearing mice treated with Taxol^®^ and PTX-loaded 4-arm-PEG_5K_-TPGS NP *in vivo* exhibited a significantly improved growth inhibitory effect as compared with that observed for the PTX-free group (57). Additionally, the EPR effect differs between different tumor types due to the protean pore dimensions in the vasculature (63). Thus, the heterogeneity of STSs and their stroma can severely impact the efficacy of passively targeted delivery. For example, doxorubicin-based chemotherapy remains the gold standard treatment for recurrent and metastatic STSs (64). Dodd et al. (58) compared the efficacy of nano-encapsulated doxorubicin and free doxorubicin in the treatment of MPNST and UPS mouse sarcoma models and observed different responses of various STS subtypes to the nanoparticle-encapsulated doxorubicin formulation (CP-Dox) treatment.

Furthermore, nanomaterials may be used not only as drug carriers but also as antitumor agents. Recently, the anticancer effect of cerium oxide nanoparticles was observed for mouse sarcoma tumor cells (59). Afterwards, *in vivo* experiments were performed by the same research team to confirm that cerium oxide nanoparticles were mainly concentrated in the mouse fibrosarcoma tissues and exhibited no apparent toxicity to the mouse liver and kidney, suggesting their potential applications in the treatment of fibrosarcoma (11). The proposed mechanism of the antitumor effect of these nanoparticles included antioxidant activity (65, 66) and passive targeting on the tumor side (67). Karuppaiya et al. reported for the first time a detailed procedure for the production of gold nanoparticles using an aqueous extract of *Dysosma pleiantha rhizome (* 60). Interestingly, the biosynthesized nanoparticles were able to inhibit the migration of the human fibrosarcoma cell line HT-1080 by interfering with the actin polymerization pathway; however, no toxic effects were observed *in vitro*. Thus, their biocompatibility and anti-migration effect may be utilized to enhance the antitumor effect of the chemotherapeutics currently used in multimodality formulations, especially in metastatic STSs. In summary, the optimal design of nanoparticles for STSs treatment is a daunting task due to the high heterogeneity and constantly evolving nature of STSs.

### Use of Nanomaterials in STS Photodynamic Therapy

Photodynamic therapy (PDT) is a tumor-ablative and disease site-specific treatment modality. It involves the generation of cytotoxic reactive oxygen species (ROS) by illuminating a photosensitizer (PS) within tumor cells with the light having a specific wavelength (68). ROS play significant roles in the physiological activities of cells at moderate concentrations. However, the overproduction of ROS may contribute to the development of many diseases, including rheumatoid arthritis (69), cardiovascular disease (70), and even COVID-19 (71). In PDT, ROS cause irreversible damage to the cells and microvasculature of solid tumors followed by a plethora of inflammatory and immune responses. Thus, the ideal PS should be capable of preferentially accumulating in tumor cells and generating a sufficient amount of ROS. Nanomaterials have recently emerged in the field of PDT to overcome most limitations of classic PSs. They may be potentially utilized in PDT as delivery vehicles for PS, PS alone, or PS energy transducers. For example, gold nanoparticles have been used to load a novel PS, N-aminobacteriopurpurinimide, for improving drug delivery through a passive targeting strategy (61). Rats bearing sarcoma M-1 demonstrated an extended circulation time in the blood and enhanced tumor uptake *in vivo*.

Moreover, a combination of different therapeutic methods with PDT may increase the antitumor efficacy (62, 72, 73). For this purpose, Luo et al. (62) introduced hydrophilic indocyanine green derivative (ICG-Der-02) into mesoporous silica-coated gold nanorods (AuNRs/mSiO_2_). They found that the nanoconjugated AuNRs/mSiO_2_–ICG-Der-02/RGD-4C system preferentially bound to HT-180 human fibrosarcoma cells and exhibited bimodal photothermal and photochemical effects under the 808-nm irradiation, thus improving the effectiveness of photodynamic therapy. In addition, a nanozyme that can increase the content of H_2_O_2_ in the tumor microenviroment may further enhance the antitumor effect of phototherapy (72). These novel multimodal treatment approaches may enhance tumor control in localized STSs.

### Use of Nanomaterials in STS Radiotherapy

Radiotherapy is one of the most promising tumor control strategies. During radiotherapy, cellular components, especially DNA, are directly or indirectly damaged by delivering ionizing radiation (IR) to tumor tissues. Increasing the radiotherapy efficacy while maintaining the normal tissue toxicity at a tolerable level remains a challenging task. Metal nanoparticles have attracted significant interest in recent years due to their promising role in enhancing the radiosensitizer effect (74). In particular, gold nanoparticles have been most extensively investigated owing to their biocompatibility and multifunctional properties (75–78). For instance, these nanoparticles were used as radiosensitizers and CT contrast agents to develop Au-loaded polymeric micelles (GPMs) in a study conducted by the Tsourkas research group (79). They found that GPMs exhibited a longer circulation half-life and six-fold accumulation in tumors. An *in vivo* study confirmed that the median survival time of the GPM-radiosensitized mice bearing human fibrosarcoma cells (HT1080) in combination with RT was 1.7 times longer than that of the mice treated with radiation alone (79).

Moreover, hafnium oxide nanoparticles NBTXR3 possess the ability to interact with different types of IR and accumulate in cancer cells, thus producing high-dose energy deposition (80). A preclinical study confirmed the promising uptake rate and encouraging radioenhancing effect of NBTXR3 in fibrosarcoma cell lines (Hs913T, HT-1080) (81). The initial phase I clinical trial of NBTXR3 activated by radiotherapy in patients with locally advanced STSs was performed by Bonvalot et al. (82) In their study, 22 patients with limb or trunk STSs were enrolled into the treatment program, and all of them received a single injection of NBTXR3 followed by external beam radiotherapy and surgical resection. The obtained results revealed that the median decrease in the maximal tumor diameter at the recommended dose was 29% with a median change in volume of −40%. This initial success led to a phase II–III trial (83) aimed to further evaluate the efficiency and safety of NBTXR3 as a radioenhancer in the preoperative treatment of patients with locally advanced STSs. A total of 176 eligible patients out of 180 enrolled ones were evaluated for the primary endpoint (pathological complete response in the intention-to-treat full analysis set). The proportion of patients with a pathological complete response exhibited a significant difference of 16% for the NBTXR3 group versus 8% for the radiotherapy alone group (p = 0.044). As a result, NBTXR3 has been approved for the treatment of STSs and became a first-in-class radioenhancer. The described tumor reduction strategy may facilitate surgical resection and provide a reference for the radiation-enhanced therapy of other solid tumors (84).

## Conclusion

STSs can occur anywhere in the body and originate from mesenchymal tissues. Overall, the patients diagnosed with STSs have poor prognosis, while STS heterogeneity represents a considerable challenge for both detection and treatment. Nanomaterials are playing increasingly important roles in STS detection and treatment. Continuing advances in research studies utilizing nanomaterials as drug delivery systems, therapeutic approaches, and their combinations can help develop an efficient and safe method for the diagnosis and treatment of STSs. However, further optimization of nanomaterials is necessary to achieve rapid diagnosis and effective treatment of STSs.

## Author Contributions

All authors contributed to the design of the study and writing of the manuscript. CZ, XC and YH undertook the research. QZ, SZ and WF wrote the main manuscript text and prepared figures. WF and SZ revised the article critically for important intellectual content and final approval of the version to be submitted. All authors reviewed the manuscript. All authors contributed to the article and approved the submitted version.

## Conflict of Interest

The authors declare that the research was conducted in the absence of any commercial or financial relationships that could be construed as a potential conflict of interest.

## Publisher’s Note

All claims expressed in this article are solely those of the authors and do not necessarily represent those of their affiliated organizations, or those of the publisher, the editors and the reviewers. Any product that may be evaluated in this article, or claim that may be made by its manufacturer, is not guaranteed or endorsed by the publisher.

## Abbreviation

STS(s), soft tissue sarcoma(s); MRI, magnetic resonance imaging; UPS, undifferentiated pleomorphic sarcoma; TKIs, tyrosine kinase inhibitors; GISTs, gastrointestinal stromal tumors; TAPs, tumor-associated proteins; DDSs, drug delivery systems; GLUTs, glucose transporters; LPR, lipid protamine-siRNA; ARMS, alveolar rhabdomyosarcoma; EPR, enhanced permeability and retention; PTX, paclitaxel; PDT, photodynamic therapy; ROS, reactive oxygen speciesp; PS, photosensitizer; IR, ionizing radiation.
